# Impact of COVID‐19 on Psychology among the University Students

**DOI:** 10.1002/gch2.202000038

**Published:** 2020-09-28

**Authors:** Bablu Kumar Dhar, Foster Kofi Ayittey, Sabrina Maria Sarkar

**Affiliations:** ^1^ Faculty of Business Daffodil International University P. O. Box 1204 Dhaka Bangladesh; ^2^ Department of Chemical Engineering Curtin University Malaysia P. O. Box 98009 Miri Sarawak Malaysia; ^3^ Young Women's Christian Association (YWCA) P. O. Box 4000 Chittagong Bangladesh

**Keywords:** COVID‐19, mental health, university students

## Abstract

The purpose of the study is to find out the psychological impact of the COVID‐19 pandemic on university students. The study focuses on the university students from different public and private universities of Bangladesh through a set of questionnaires according to the guideline of Generalized Anxiety Disorder Scale (GAD‐7). The result among 15 543 respondents shows that 44.59% are suffering from severe anxiety, 48.41% moderate anxiety, and only 3.82% mild anxiety. The results highlight that all epidemic‐related stressors are positively correlated with the level of anxiety. Among the epidemic‐related stressors, worry about economic influences during and after COVID‐19 (*r* = 0.342, *p* < 0.001) and worry about the influence of COVID‐19 on daily life (*r* = 0.340, *p* < 0.001) have a highly positive impact on the level of anxiety. Following these stressors, worry about academic delays due to COVID‐19 (*r* = 0.326, *p* < 0.001) and worry about the social support during COVID‐19 (*r* = 0.321, *p* < 0.001) have moderately and positively correlated with the level of anxiety. The study suggests that proper government support, as well as social awareness, should be monitored during epidemics for decreasing anxiety and maintaining a good mental health of the university students.

## Graphical abstract

The purpose of this study is to evaluate the psychological impact of the COVID‐19 pandemic among university students in Bangladesh. Through cluster sampling and a set of questionnaires based on Generalized Anxiety Disorder Scale (GAD‐7), the study attempts to determine the association. The results show that 44.59% are suffering from severe anxiety, 48.41% moderate anxiety, and 3.82% mild anxiety.

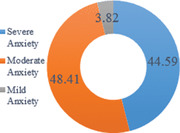

## 1 Introduction

In 2020, the current population density of Bangladesh is 1115.55 people per square kilometer, which is a 1.01% increase than last year. Because of the density of population, Bangladesh is the 12th most densely populated country in the world.^[^ ^1^ ^]^ According to the report of the World Economic Forum, COVID‐19 threatens to cause a humanitarian crisis in this country. According to the World Bank data, in Bangladesh, 15% of workers earn more than 500 taka ($5.90) a day and the economic shutdown sparked by COVID‐19 endangers millions of livelihoods imminently. The people could meet their daily expenditures, sent their children to school, and expected that they could save money for an emergency health crisis. The majority of villagers depend on remittances from the cities or overseas. However, for the current global crisis, people are out of work. Income has halted.

Based on the report of the Reuters, the COVID‐19 pandemic was confirmed to have spread to Bangladesh on March 2020 after recognizing the first three known cases reported on March 7, 2020, by the Institute of Epidemiology, Disease Control and Research (IEDCR) of the country.^[^ ^2^ ^]^ According to IEDCR, Bangladesh, the level of infections remained low at the end of March, although there was a precipitous rise in April. In the week ending April 16, new cases in Bangladesh grew 1155%, the highest in Asia, ahead of Indonesia with 186%. As of June 3, 2020, there are a total of 57 563 confirmed cases besides 12 161 recovered and 781 death cases.

The novel coronavirus (COVID‐2019) is spreading faster all over the country. The current situation imposes massive pressure on the government of Bangladesh, the general public, and healthcare, and medical providers.^[^ ^3^ ^]^ The pandemic has brought not only the danger of death from the epidemiologic contagion but also an intolerable psychological burden to the people among the world^[^ ^4^, ^5^ ^]^ and Bangladesh is not out of the problems. The faster spread of the virus; strict isolation measures; and delays in starting schools, colleges, and universities across the country are anticipated to influence the psychology of university students.^[^ ^6^ ^]^ Psychological reports indicate the influence of the epidemic on the children, public, older adults, medical staff, and patients.^[^ ^7^, ^8^, ^9^ ^]^ However, no thorough research on the psychology of university students facing the epidemic has been conducted to date.

Like other sectors, the education sector has been affected severely in Bangladesh. On March 17, 2020, the government closed all schools, colleges, and universities when Bangladesh had eight confirmed cases. Among 853 267 students out of 104 universities in Bangladesh, the number of public universities is 33 with 454 530 students, and the number of private universities is 71 with 398 737 students. The average student count for a public university is 13 774, and 5616 for a private university that indicates that there are 8204 students per university. Having a vast number of undergraduate, graduate, and postgraduate students, the country wishes a secure future that has been temporary collapsed due to the current pandemic.

For detecting anxiety disorders, the seven‐item Generalized Anxiety Disorder Scale (GAD‐7) is one of the extensively used tools that is easy to score and takes less than 1–3 min to finish.^[^ ^6^ ^]^ Moreover, the GAD‐7 is also applicable for diagnosis, screening, and the valuation of the strictness of anxiety disorders, and also for panic disorders, stress disorders, post‐traumatic disorders, and social phobia.^[^ ^10^ ^]^

The prior problem of the students of university is mostly related to their career and future. Due to the uncertainty, stress increases among university students;^[^ ^11^ ^]^ hence, the method of supervisory university students during public health crises is different and challenging. Therefore, the study aimed to analyze the psychological impact of COVID‐19 pandemic among university students in Bangladesh. Moreover, the study intends to provide suggestions to the government and non‐government organizations for taking the necessary steps.

## 2 Results

### 2.1 Demographic Statistics

**Table** **1** shows the demographic and particular characteristics of the population of the study. The table indicates the 15 543 university students, most of the respondents are male (66.7%), approximately two‐thirds of them live in the urban area (76.1%), most of their financial status is not steady (61.6%), majority of the participants live with their parents (78.1%), and many of their relatives or friends are not infected with COVID‐19 (87.4%). However, 12.6% of the relatives or friends of the respondents have been infected by the virus.

**Table 1:** Demographic profile of the respondents

	Frequency	Percent
Gender		
Male	10 395	66.7%
Female	5148	33.3%
Place of residence		
Urban	11 880	76.1%
Rural	3663	23.9%
Financial condition		
Steady	9504	61.6%
Not steady	6039	38.4%
Living status		
Live with parents	12 177	78.1%
Live without parents	3366	21.9%
Relatives or friends infected with COVID‐19		
Infected	1881	87.4%
Not infected	13 662	12.6%

### 2.2 Anxiety Level among University Students during the Outbreak

**Table** **2** demonstrates the psychological health of university students who have been affected during the epidemic. It is shocking to observe that most of the students are at a high level of anxiety, and their psychological condition is alarming. Among 15 543 students, a very few number of students are in normal (3.18%) and mild (3.82%) level of anxiety. However, nearly half of the students are at a moderate level of anxiety (48.41%), and the rest of them are suffering from very high or severe level (44.59%) of anxiety.

**Table 2:** Different anxiety level among the number of university students (*n* = 15 543)

Level of anxiety	Number of students	Ratio [%]
Normal	495	3.18
Mild	594	3.82
Moderate	7623	48.41
Severe	7326	44.59

### 2.3 Influencing Factors of Anxiety among University Student during the Outbreak

#### 2.3.1 Univariate Analysis

The association between the demographic variables and the level of nervousness or anxiety among Bangladeshi university students are pointed in **Table** **3**. The analysis shows that all factors have a significant effect on anxiety. During the epidemic, gender has a significant effect on anxiety (*p* < 0.05) and males are severely worried (16.88%) than female during the epidemic; place of residence in the urban area has a significant impact on anxiety and living in the urban area creates moderate anxiety (18.79%) than rural areas; living with their parents has a substantial consequence on anxiety and students who live alone has amplified anxiety level (*p* < 0.05). On the other hand, financial condition, and the infection of COVID‐19 among relatives or friends have the most significant effect on anxiety (*p* < 0.001).

**Table 3:** Univariate analysis of anxiety of university students about the outbreak

Variables	Total	Level of anxiety				Statistics	*p*
		Normal	Mild	Moderate	Severe		
Gender						0.925^b)^	0.01
Male	10 395 (33.44)	99 (0.32)	495 (1.59)	4554 (14.65)	5247 (16.88)		
Female	5148 (16.56)	0 (0.00)	99 (0.32)	2970 (9.55)	2079 (6.69)		
Place of residence						2.922^a)^	0.002
Urban	11 880 (38.2)	495 (1.59)	495 (1.59)	5841 (18.79)	5049 (16.24)		
Rural	3663 (11.8)	0 (0.00)	99 (0.32)	1683 (5.41)	1881 (6.05)		
Financial condition						5.420^a)^	<0.001
Steady	9504 (30.57)	297 (0.96)	495 (1.59)	5049 (16.24)	3663 (11.78)		
Not steady	6039 (19.43)	198 (0.64)	99 (0.32)	2475 (7.96)	3267 (10.51)		
Living with parents						0.970^b)^	0.012
Yes	12 177 (39.17)	297 (0.96)	396 (1.27)	1386 (4.46)	1584 (5.10)		
No	3366 (10.83)	198 (0.64)	198 (0.64)	6318 (19.75)	5346 (17.20)		
Relatives/friends infected with COVID‐19						7.780^b)^	<0.001
Yes	1881 (6.05)	99 (0.3)	99 (0.4)	891 (2.9)	891 (2.87)		
No	13 662 (43.95)	396 (1.3)	495 (1.6)	6633 (21.3)	6039 (19.43)		

^a)^ Kruskal‐Wallis test;

^b)^ Mann‐Whitney test.

#### 2.3.2 Ordinal Regression Analysis

**Table** **4** points out the consequences of ordinal multivariate analysis of related influences with anxiety level through the epidemic among the university students of Bangladesh. Significant influences from the univariate analysis comprised in the analysis of ordered logistic regression (OLR) in the table. The model test indicates that the value of odds ratio (OR) of all variables was statistically significant (*p* < 0.05). Moreover, the chi‐square test (χ^2^ = 14.170) of the observed values indicates a good model fit. Results from the OLR analysis of the factors that influence anxiety level among university students indicate that “living in urban areas” causes more anxiety (OR = 1.940; CI = 1.830, –2.740); unstable financial condition causes more worry in comparison with stable financial condition (OR = 2.920; CI = 1.910, –3.540); living without parents increases nervousness level (OR = 1.270; CI = 0.850, 1.730); and infected relatives or friends with novel coronavirus enhance anxiety's risk factor (OR = 3.750; CI = 2.950, –3.980).

**Table 4:** OLR analysis of factors that influence anxiety level among students

Factors	Total	SE	OR	*p*	OR (95% CI)
Place of residence					
Urban	11 880	0.097	1.940	<0.001	(1.830, 2.740)
Rural	3663	0.076	0.895	0.002	(0.850, 1.110)
Financial condition					
Steady	9504	0.070	0.750	0.003	(0.710, 0.920)
Not steady	6039	0.110	2.920	<0.001	(1.910, 3.540)
Living with parents					
Yes	12 177	0.160	0.720	0.004	(0.640, 0.771)
No	3366	0.980	1.270	<0.001	(0.850, 1.730)
Relatives/friends infected with COVID‐19					
Yes	1881	0.150	3.750	<0.001	(2.950, 3.980)
No	13 662	0.060	0.725	0.002	(0.640, 0.810)

*SE* = Std. Error, *OR* = Odds ratio, *CI* = Confidence interval.

#### 2.3.3 Correlation between the Level of Anxiety and Epidemic‐Related Stressors

**Table** **5** indicates the result of the correlation analysis between the level of anxiety and COVID‐19 epidemic‐related stressors, including worry about economic influences, worry about academic delays, worry about the influence of COVID‐19 on daily life, and worry about the social support during COVID‐19. The results highlight that all epidemic‐related stressors are positively associated with the level of anxiety. Among the epidemic‐related stressors, worry about economic influences during and after COVID‐19 (*r* = 0.342, *p* < 0.001) and worry about the influence of COVID‐19 on daily life (*r* = 0.340, *p* < 0.001) have a highly positive impact on the level of anxiety level. Following these stressors, worry about academic delays due to COVID‐19 (*r* = 0.326, *p* < 0.001) and worry about the social support during COVID‐19 (*r* = 0.321, *p* < 0.001) are moderately and positively connected with the level of anxiety.

**Table 5:** Analysis of correlation between the epidemic‐related stressors and university students’ anxiety

Stressors	Anxiety level	
	*r*	*p*
Worry about economic influences during and after COVID‐19	0.342	<0.001
Worry about academic delays due to COVID‐19	0.326	<0.001
Worry about the influence of COVID‐19 in daily life	0.340	<0.001
Worry about social support during COVID‐19	0.321	<0.001

*r* = Correlation coefficient.

## 3 Discussion

According to previous studies, public health emergencies have several psychological effects on students who study in higher educational institutions. According to Cornine,^[^ ^12^ ^]^ college students’ anxiety is connected to the consequence of the virus. Bao et al.^[^ ^13^ ^]^ mentioned that the growing number of infected and suspected patients has increased anxiety level among students. Ayittey et al.^[^ ^14^, ^15^ ^]^ highlighted the significant scarcity of masks and sanitizers, and the devastating, astonishing, and erroneous news reports in different social media had increased anxiety and fear. The study of Cao et al.^[^ ^6^ ^]^ pointed that the anxiety among college students during the epidemic was related with their place of residence, source of parental income, whether living with parents, and whether a relative or an acquaintance was infected with the epidemic without significant difference in gender or region that is dissimilar from the conclusions of Moreno et al.^[^ ^10^ ^]^ Cao et al.^[^ ^6^ ^]^ concluded that male and female students have similar stresses and negative emotions as a result of COVID‐19. The study of Mei at al.^[^ ^16^ ^]^ indicated fear and anxiety. Elmer et al.^[^ ^17^ ^]^ and Wang et al.^[^ ^18^ ^]^ focused on future employment. Cornine^[^ ^12^ ^]^ emphasized on infection of relatives or friends during the epidemic. Kmietowicz^[^ ^19^ ^]^ and Xiao^[^ ^5^ ^]^ highlighted the psychological condition during long interpersonal communication.

The foremost intention of the current study was to assess the psychological situation of university students during the current epidemic, COVID‐19, and to explore the influencing factors of their anxiety. The study found that almost 97% of the university students were experiencing anxiety due to the outbreak of the current epidemic. Among the students who participated, 48.41% are suffering from a moderate level of anxiety, and 44.59% are experiencing a severe level of anxiety. On the other hand, the amount of experiencing a mild level of anxiety (3.82%) and not feeling anxiousness (3.18%) is very poor in ratio. This current study shows an opposite scenario of the study of Cao et al.^[^ ^6^ ^]^ The study of Cao et al.^[^ ^6^ ^]^ highlighted the psychological condition of college students of China during COVID‐19. The study of Cao et al.^[^ ^6^ ^]^ indicated that among Chinese students only 0.9% experienced severe anxiety and 21.3% experienced mild anxiety during the COVID‐19 outbreak.

The economy of urban areas is relatively significant and delivers citizens with better safety,^[^ ^20^ ^]^ and living in urban areas is a protective factor against anxiety.^[^ ^6^ ^]^ There is indeed an inequity of cultural, economic, and education between rural and urban areas. For example, hygienic conditions in urban areas are healthier than rural, which reduces the chances of surviving and spreading of COVID‐19.^[^ ^21^ ^]^ However, the univariate analysis and OLR analysis of factors on the level of anxiety among the students of universities in Bangladesh show a different scenario. In explanation, the participants mentioned the reason of overdensity among the urban areas in Bangladesh. Most of the higher educational institutions are in urban areas. Due to the reason, a majority of the students live in urban areas and experience immense anxiety during the current epidemic.

Living without parents was another favorable factor for increasing anxiety among students. The current study also finds a similar result regarding this factor with the study of Cao et al.^[^ ^6^ ^]^ Earlier studies also have specified that the risk factors connected with anxiety and emotional sicknesses among adults comprise not living with parents, parents’ physical and psychological problems, and the death of parents in infantile,^[^ ^22^ ^]^ which are reliable with the results of the present study.

Financial stability or condition matters much on anxiety among the students.^[^ ^6^ ^]^ Elmer et al.,^[^ ^17^ ^]^ also found that the constancy of family income has a significant influence on the anxiety level of the university students during the COVID‐19 catastrophe.

Higher levels of stress are connected with young age, having to go out to work, female gender, and having an acquaintance infected with COVID‐19.^[^ ^23^ ^]^ The contemporary study also found that having relatives or friends being infected with novel coronavirus becomes a risk factor among university students’ anxiety during the epidemic. According to the respondents, it generates high contagiousness of the COVID‐19.^[^ ^24^ ^]^

Economic stressors, academic delays, effects on daily life, and families or friends being infected with epidemic are positively related to anxiety among the university students of Bangladesh during the epidemic. Similar studies by Cao et al.^[^ ^6^ ^]^ have specified that along with the national health condition, COVID‐19 also has a substantial influence on the economy of the country as well as individuals.^[^ ^25^ ^]^ Bangladesh remains on a knife‐edge in COVID‐19 crisis. The lockdowns prompted by the epidemic has already deteriorated the economic and political stability of the country. Due to the outbreak, many families are losing their source of income, and students are feeling worried about paying their tuition fees.^[^ ^26^ ^]^ Hunger, malnutrition, and other related problems have augmented in Bangladesh as a result of the lockdown.^[^ ^26^ ^]^ Like other countries, all primary, secondary schools, high schools, colleges, and universities were closed and delaying classes until March 17, 2020, which creates more anxiety among students. For reducing anxiety regarding this issue, using distant/remote learning methods can be a good model. These actions certainly have a precise influence on education and the development of students.

Social support is also positively correlated with the anxiety of the university students of Bangladesh, which is not consistent with previous findings.^[^ ^6^, ^7^ ^]^ According to the participants of the study, social support lessens the mental pressure during the epidemic and changes the attitude regarding social support that is rare to find in the society by the students. The social support in Bangladesh is not run under a single umbrella like the developed countries. Even the supports from the government are not well enough.^[^ ^27^ ^]^ This consequence indicates that active and vigorous social support is essential during public health emergencies to reduce the anxiety level among the students.

## 4 Conclusions

Being burdened with population, Bangladesh becomes one of the major risky zones during COVID‐19 epidemic where financial, physical, and psychological crisis arise in every moment. The overall situation creates a deep psychological impact among the university students of Bangladesh. About 97% of university students are in deep anxiety due to the current epidemic. Living in urban areas, not having a steady financial situation, not living with parents, and infection of relatives or friends in epidemic become the severe factors for severe anxiety among university students during the outbreak of novel coronavirus. The stressors of the COVID‐19, including economic stressors, academic delays, impact on daily life, and social supports, are entirely linked with the symptoms of anxiety levels among the university students of Bangladesh during the epidemic. Though the government of Bangladesh is adopting several policies regarding this issue, consciousness and preventive measurements of the inhabitants of the country according to the guidelines of World Health Organization are needed more to resolve the critical problem with high priority. Proper government support, as well as social awareness, should be monitored during epidemics to decrease anxiety and maintaining the good mental health of the university students for crafting a better future of the nation.

## 5 Experimental Section

### Study Population and Sample

For the analysis, the study targeted the public and private university students of Bangladesh. The respondents were selected randomly from different universities in different cities in Bangladesh. By using a structured, reliable, and confidential questionnaire, the study tried to measure the psychological health of the university students during the COVID‐19 outbreak. A total number of 15 543 respondents responded (100% response rate) willingly for discovering the psychological effect of the COVID‐19 pandemic among university students in Bangladesh.

### Instruments

For finding out the psychological impact on the university students of Bangladesh, the study used GAD‐7 that comprises seven items constructed on seven core symptoms and queries to find out the respondents who suffered within the last 2 weeks.^[^ ^28^ ^]^ The total score range of the questions was 0–21 followed by a four‐point Likert scale from 0 to 3 (not at all to almost every day).^[^ ^6^ ^]^ Moreover, the study tried to find out the related demographic information of the respondents, including gender, place of residence, financial situation, living condition, and status of the infection of COVID‐19 among their relatives or friends. Furthermore, the respondents were queried about their thoughts and preventive behaviors, economic conditions, academic progress, availability of social support, and influence on daily life during the epidemic. The internal consistency (Cronbach's ɑ) of GAD‐7 is 0.929.

### Data Analysis

SPSS (version 25.0) was used to analyze the collected data. The study implemented several statistical methods to justify the impact of COVID‐19 epidemic among the Bangladeshi university students, including descriptive statistics to demonstrate the demographic features; univariate analysis (nonparametric test) to discover the significant relations between sample characteristics and anxiety level;^[^ ^29^ ^]^ multivariate logistic regression analyses to determine statistical significance among the variables through odds ratio with 95% confidence of interval;^[^ ^6^ ^]^ Spearman's correlation coefficient (*r*) with a two‐tailed statistical significance (*p* < 0.05) to assess the connotation between novel coronavirus‐related stressors and the level of anxiety.

### Ethical Considerations

The department of students’ affairs of different universities approved this study. The university authorities also felt interested to know the psychological impact of COVID‐19 epidemic on the students. After describing the need of the study, respondents gave their consent voluntarily.

## Conflict of Interest

The authors declare no conflict of interest.
